# Conversion of 5-hydroxymethylfurfural to furan-2,5-dicarboxylic acid by a simple and metal-free catalytic system†

**DOI:** 10.1039/d3ra01104f

**Published:** 2023-05-10

**Authors:** Xue Wang, Xinyuan Guo, Xinmei Wang, Chi Li, Shanjun Wang, Han Li, Yan’an Gao, Yiying Li, Jinhui Wang, Huanjun Xu

**Affiliations:** a Department of Medicinal Chemistry and Natural Medicine Chemistry, College of Pharmacy, Harbin Medical University Harbin China 15798946232@163.com; b School of Science, Qiongtai Normal University Haikou 571127 China; c Key Laboratory of Ministry of Education for Advanced Materials in Tropical, Island Resources, Hainan University Haikou 570228 China; d College of Basic Medicine and Life Sciences, Hainan Medical University Haikou China; e Key Laboratory of Child Cognition and Behavior Development of Hainan Province, Qiongtai Normal University Haikou China

## Abstract

A simple and metal-free catalytic system composed of NaO^*t*^Bu/DMF and an O_2_ balloon efficiently converted 5-hydroxymethylfurfural (5-HMF) to furan-2,5-dicarboxylic acid with an 80.85% yield. 5-HMF analogues and various types of alcohols were also transformed to their corresponding acids in satisfactory to excellent yield by this catalytic system.

## Abstract 2

A simple and metal-free catalytic system composed of NaO^*t*^Bu/DMF efficiently converted 5-HMF to FDCA with an 80.85% yield. 5-HMF analogues and various types of alcohols were also transformed to corresponding acids in satisfactory to excellent yield.

## Graphical abstract

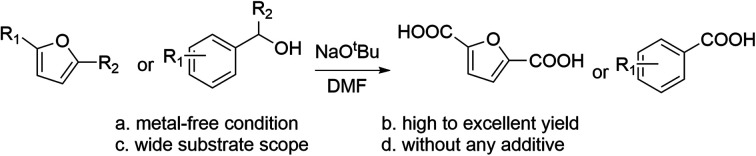

Biomass is a renewable and inexpensive resource with wide availability and high biodegradability.^1^ Thus, there is practical significance for all the monomers derived from biomass according to green and sustainable chemistry. Abundant resources, such as C_6_ carbohydrates, can be used to prepare 5-hydroxymethylfurfural (5-HMF).^2^ It is well known that carbohydrates (cellulose) can be converted to 5-HMF through the hydrolysis of cellulose to glucose, the isomerization reaction of glucose to obtain fructose, and the subsequent removal of three molecules of water by fructose to obtain 5-HMF derivatives.^3^

5-HMF is an intermediate used to obtain pharmaceutical ingredients and important useful bio-based acids, aldehydes, alcohols, and amines.^4^ The derivative furan-2,5-dicarboxylic acid (FDCA) is considered to be one of the most relevant bio-based building blocks.^5^ FDCA has been used as a material from which poly(ethylene 2,5-furandicarboxylic acid) is obtained, which is the most promising alternative to replace poly(ethyleneterephthalate) (PET) in the market, due to its sustainability and satisfactory barrier properties.^6^

There has been considerable research on the synthesis of FDCA from 5-HMF. It was shown that metal-mediated homogeneous and heterogeneous catalysis shared the target to achieve FDCA in satisfactory yield under relatively mild conditions. In conventional processes, there are practical applications for catalysts based on Au,^7^ Pt,^8^ Pd,^9^ Ru,^10^ Ag,^11^ Mn,^12^ Co,^13^ Fe,^14^ and Mo^15^ in the production of FDCA. However, according to the sustainability perspective and green chemistry, it is necessary to avoid the usage of toxic reagents and metal-based catalysts when biomass is used as a feedstock.

To overcome the problems related to the use of metal-based catalysts for the direct oxidation of 5-HMF to FDCA, such as high cost of noble metal catalysts and their leach-inducing environmental contamination, there has been great interest in metal-free catalysis. However, there have been few research studies on sustainable, cost-effective, metal-free alternatives (Table S1†) for this reaction. Most of them are heteroatom-doped (N, P, and S) carbon-based catalysts. Nguyen *et al.* synthesized a porous nitrogen-doped metal-free nano-carbon catalyst, NNC-900. When NNC-900 catalyzed HMF under normal pressure at 80 °C, a yield of 80% FDCA was obtained.^16^

Carbon-based selenium-containing materials, such as carbohydrate-supported selenium, selenium-doped carbon (Se/C), and selenium-doped polymeric carbon nitride (PCN-Se) can be applied in biomass transformation.^17^ In 2017, Elias *et al.* reported the I_2_/NaOH-catalyzed oxidation of 5-HMF to FDCA using water as the solvent and TBHP as the co-oxidant.^18^ These catalysts are sometimes very effective, but may require a tedious preparation process or multi-component mixture, long reaction time, high-boiling solvent, and a large excess of oxidant (Table S1†).

Herein, we report a very efficient approach to achieve the oxidation of HMF to FDCA based on NaO^*t*^Bu/DMF with an oxygen balloon. In previous reports, NaO^*t*^Bu–O_2_ oxidatively cleaved vic-1,2-dios to carboxylic acid^19^ and allylic alcohols to α,β-unsaturated carboxylic acids.^20^ The NaO^*t*^Bu/DMF catalytic system exhibited a nearly 100% conversion of HMF, with 80.85% yield of FDCA at 45 °C for 6 h. Initially, the reaction was conducted under 45 °C and 4 equiv. NaO^*t*^Bu in DMF for various reaction times. When this reaction was conducted for 0.5 h, the conversion of 5-HMF was 99.63%, with 5-hydroxymethyl-2-furancarboxylic acid (HMCFA) at 49.19% and FDCA at 35.65% being obtained (Table S1,† entry 1 and Table S1,† entry 2, respectively).

It was clearly shown that the reaction proceeded very quickly at the beginning, with an FDCA yield of 72.38% after 2 h. After the reaction time was extended to 6 h, the yield of FDCA only increased to 77.90%. Additionally, in this reaction, an unknown product existed throughout the entire reaction process, and the yield of FDCA was increased, with a decrease in the yield of HMCFA (Table S2†). Next, the amount of the catalyst was examined, giving rise to 80.85% yield of FDCA when NaO^*t*^Bu (5 eq.) was used. It was clear that the yield of FDCA increased with increasing amounts of NaO^*t*^Bu. However, there was little decrease in the product yield when the amount of NaO^*t*^Bu was increased to 6 eq. That is, in this reaction, the yield of product cannot be significantly increased with the use of a catalyst amount of up to 5 eq.

We further examined the reaction temperature in an attempt to increase the yield of FDCA. Unfortunately, when the reaction temperature was increased to 55 °C and 60 °C, the yield of FDCA decreased instead. Next, various bases were tested, and usage of LiO^*t*^Bu, KO^*t*^Bu, KOH, NaOH, K_2_CO_3_, Na_2_CO_3_, NaHMDS, and DBU resulted in FDCA yields of 8.34%, 28.75%, 1.20%, 40.23%, 0%, 0%, and 0%, respectively. Interestingly, when *tert*-butoxide ions were used, the chemical yield of FDCA increased in the following order: NaO^*t*^Bu > KO^*t*^Bu > LiO^*t*^Bu, which implied that the Na^+^ ion might play an important role in this transformation. It was previously reported that the cause might be related to whether each base dissolves as an ion pair or free ions in DMF.^21^

NaO^*t*^Bu dissolves in some solvents as an ion pair, although the interaction must be weaker than that of LiO^*t*^Bu due to the moderate positive charge density of Na^+^, and the ion pair must be able to gradually progress to free ions. KO^*t*^Bu dissolves in DMF mostly as free ions.^19,20^ With the increase in the cation radius, the salt ionic property increased, thus resulting in enhanced solubility. These phenomena cannot be fully explained, but may be dependent on whether each base dissolves in DMF as an ion pair or free ions, which further prompts the catalytic system basicity to adjust this oxidation process. When the cation was Na^+^ ions, *tert*-butoxide > OH^−^ > HMDS^−^ > CO_3_^2−^. Strong bases such as NaHMDS, DBU, NaOH, and KOH resulted in a lower yield or no yield of the desired product.

These results indicated that cations and anions play a crucial role in the reaction. Apart from the cation, the influence of the anion on the reaction was not negligible, mainly with respect to its basicity that is positively correlated with nucleophilicity. In this reaction, *tert*-butoxide showed strong synergistic effects and unprecedented activity in this oxidation system, indicating that it could serve as a valuable complement to transition metal catalysts. Among those with a *tert*-butoxide base, NaO^*t*^Bu as the catalyst was superior as compared to other bases.

Lastly, we studied the effect of solvents on the reaction. For all of the solvents, the yield of product in DMF was superior to others, while there was less yield (DMSO) or no yield (EtOAc, MeOH, toluene) with other solvents, which might be due to the poor solubility in some solvents (such as EtOAc and toluene). Additionally, the donor–acceptor properties of DMF might be higher than those of other solvents. Some researchers have demonstrated the synergetic role of *tert*-butoxide base and DMF for the initiation of the radical process,^22–24^ in which experimental and computational studies provided unambiguous evidence that DMF is an active species in the initiation step.

When this reaction proceeded under N_2_ or open air, the product was not obtained, or there was a relatively low yield (entries 25 and 26). We achieved the oxidation of 5-HMF to FDCA using a very simple catalyst system of NaO^*t*^Bu/DMF with an oxygen balloon. The catalytic system exhibited nearly 100% conversion of 5-HMF, with 80.85% yield of FDCA under 45 °C for 6 h (Table 1).

**Table 1:** Optimization of reaction conditions^a^

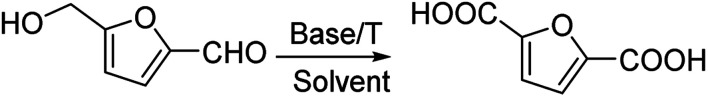						
Entry	Base	*T*/h	Temp./°C	Solvent	Yield (%)	Selectivity (%)
1	NaO^*t*^Bu	0.5	45	DMF	36.56^b^	36.60
2	NaO^*t*^Bu	1	45	DMF	47.74^b^	35.69
3	NaO^*t*^Bu	2	45	DMF	72.38^b^	72.46
4	NaO^*t*^Bu	3	45	DMF	76.20^b^	76.31
5	NaO^*t*^Bu	6	45	DMF	77.90^b^	77.97
6	NaO^*t*^Bu	9	45	DMF	77.32^b^	77.50
7	NaO^*t*^Bu	6	45	DMF	51.40^c^	51.62
8	NaO^*t*^Bu	6	45	DMF	73.45^d^	74.18
**9**	**NaO** ^***t***^ **Bu**	**6**	**45**	**DMF**	**80.85**	**80.89**
10	NaO^*t*^Bu	6	45	DMF	80.08^e^	80.08
11	NaO^*t*^Bu	6	35	DMF	72.92	73.05
12	NaO^*t*^Bu	6	55	DMF	70.61	71.12
13	NaO^*t*^Bu	6	65	DMF	64.02	64.59
14	LiO^*t*^Bu	6	45	DMF	8.34	14.16
15	KO^*t*^Bu	6	45	DMF	28.75	28.75
16	KOH	6	45	DMF	1.20	5.91
17	NaOH	6	45	DMF	40.23	44.62
18	NaHMDS	6	45	DMF	—	—
19	DBU	6	45	DMF	—	—
20	K_2_CO_3_	6	45	DMF	—	—
21	Na_2_CO_3_	6	45	DMF	—	—
22	NaO^*t*^Bu	6	45	DMSO	45.32	52.64
23	NaO^*t*^Bu	6	45	EtOAc	—	—
25	NaO^*t*^Bu	6	45	MeOH	—	—
25	NaO^*t*^Bu	6	45	Toluene	—	—
26	NaO^*t*^Bu	6	45	DMF	—f	—
27	NaO^*t*^Bu	6	45	DMF	70.21^g^	70.28

a Conditions: substrate (0.5 mmol), base (2.5 mmol), solvent (5 mL), O_2_ balloon, HPLC yield.

b NaO^*t*^Bu (2.0 mmol).

c NaO^*t*^Bu (1 mmol).;

d NaO^*t*^Bu (1.5 mmol).

e NaO^*t*^Bu (3 mmol).

f N_2_ balloon.

g Open air.

After determining the optimized reaction conditions, the substrate scope and limitation of this catalytic system were explored. As depicted in Table 2, various 5-HMF analogues were first treated with NaO^*t*^Bu/DMF. 5-Hydroxymethyl-2-furancarboxylic acid (HMFCA), 5-formylfuran-2-carboxylic acid (FFCA), and furan-2,5-dicarbaldehyde (DFF), which were all derived from HMF, were successfully transformed to FDCA in 92.57%, 87.94%, and 85.02% yield, respectively (Table 2, entries 1–3).

**Table 2:** Catalytic aerobic oxidation of HMF analogues and various alcohols^a^

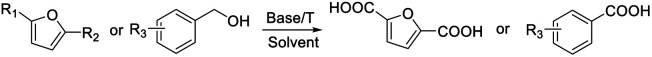			
Entry	Substrate	Product/yield^b^	
1	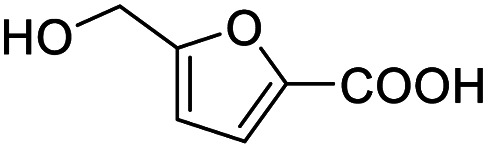	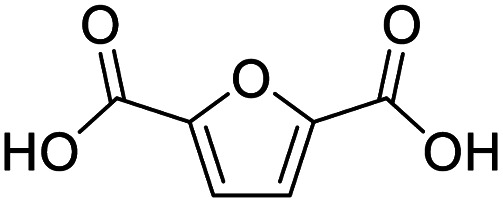	92.57^c^
2	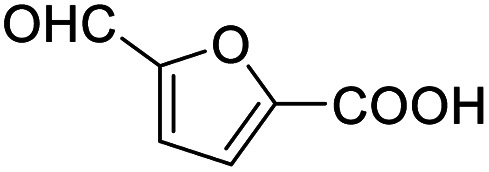	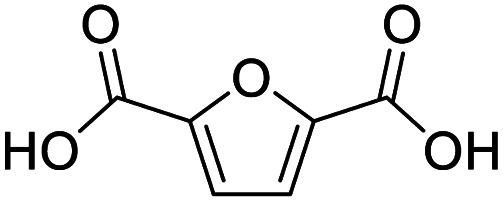	87.94^c^
3	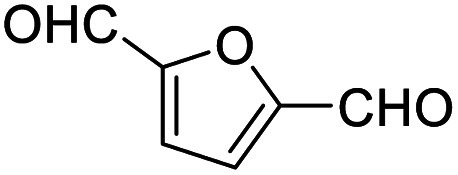	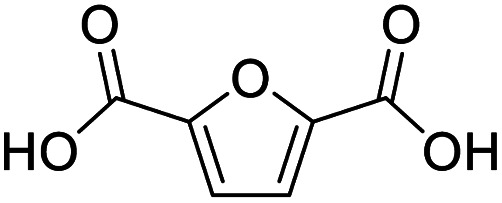	85.02^c^
4	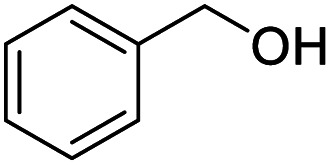	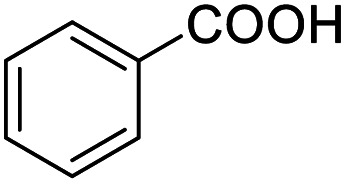	99.92
5	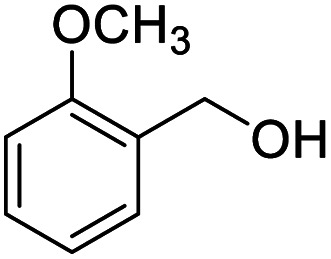	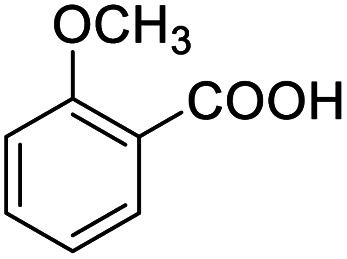	54.02
6	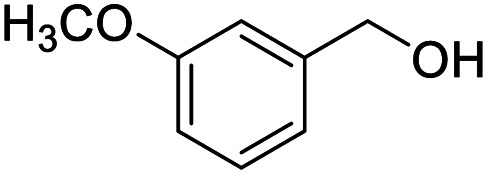	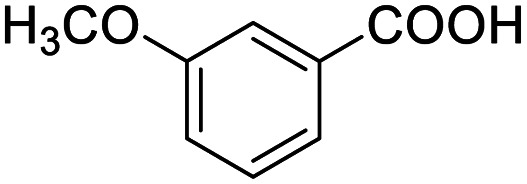	96.92
7	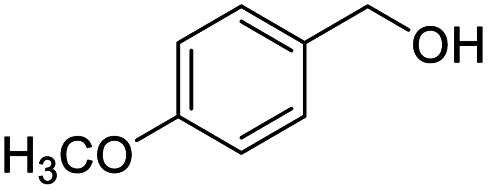	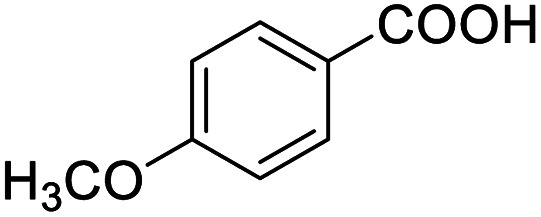	95.00
8	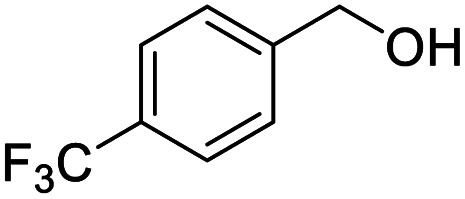	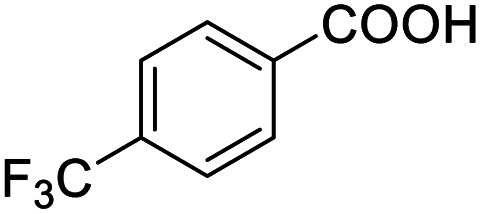	99.90
9	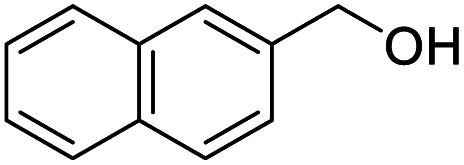	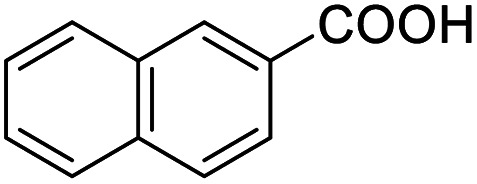	78.65
10	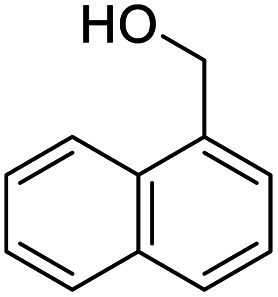	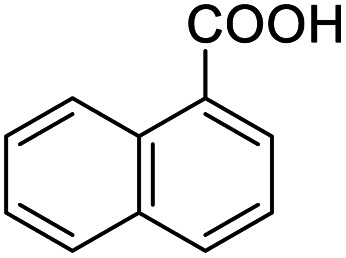	93.08
11	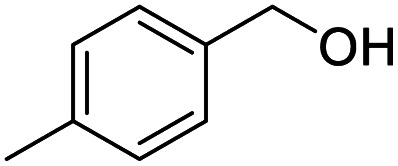	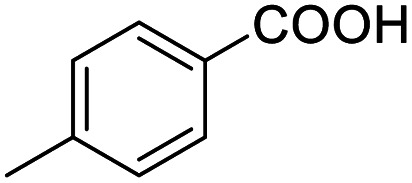	81.80
12	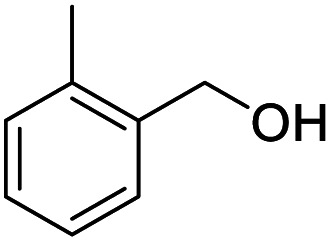	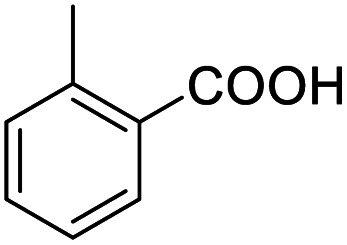	69.03
13	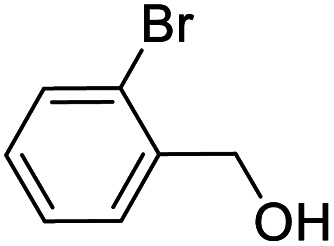	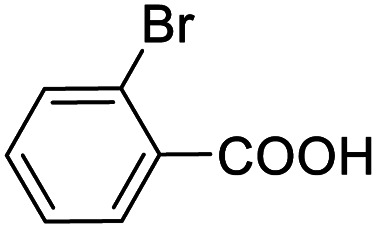	75.25
	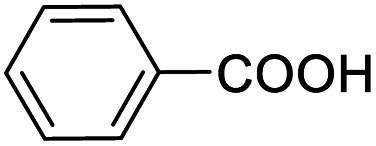	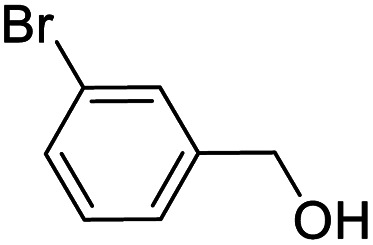	21.71
14	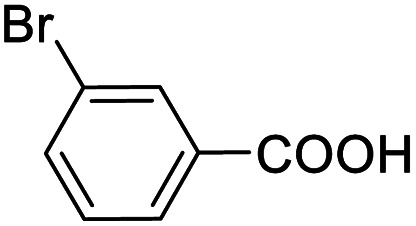	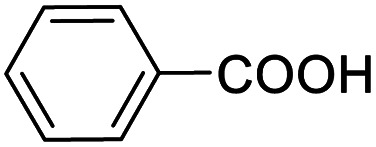	79.29
			15.53
15	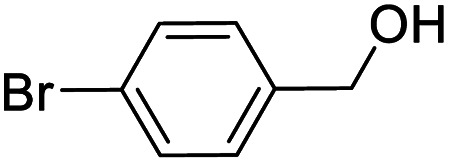	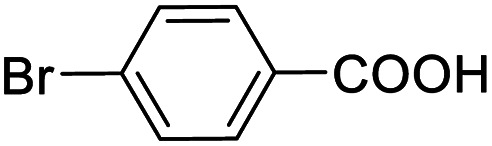	65.18
		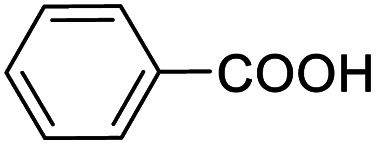	39.83
16	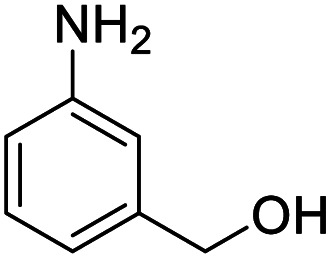	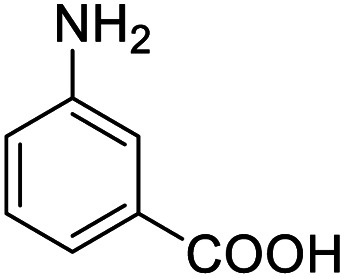	76.26
17	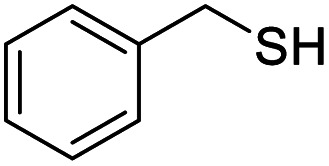	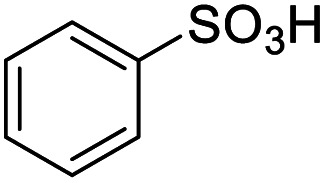	0.00
18	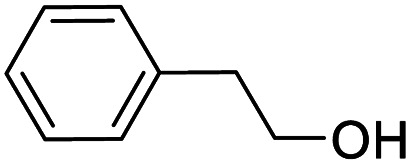	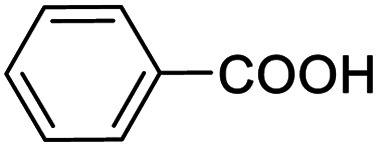	69.51
19	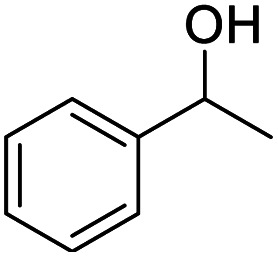	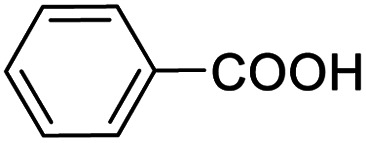	78.66
20	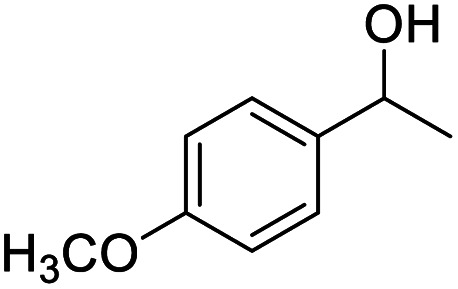	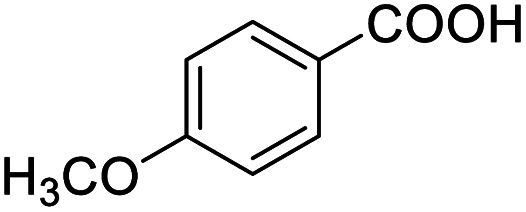	85.51
21	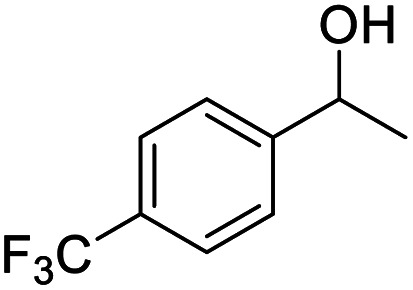	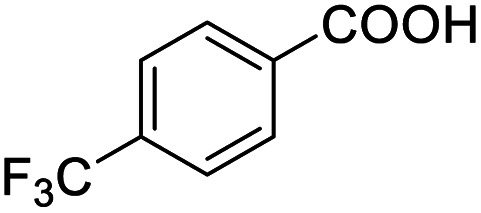	95.78
22	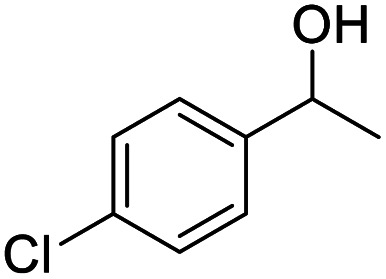	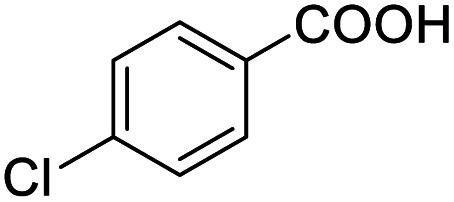	79.28

a Conditions: substrate (0.5 mmol), NaO^*t*^Bu (2.5 mmol), DMF (5 mL), O_2_ balloon.

b GC yield.

c HPLC yield.

Next, the scope of benzyl alcohols was investigated (Table 2, entries 4–10). The corresponding benzoic acids were obtained in satisfactory to excellent yield with electron-withdrawing or electron-donating groups. From the results of 2-CO_3_-, 3-CO_3_-, and 4-OCH_3_-substituted substrates, a lower yield of 2-OCH_3_-substituted substrates was obtained, which might be due to the *ortho*-effect, with 54.02% yield of product. A similar result was obtained with the –CH_3_-substituted substrate (Table 2, entries 11 and 12). The analogue with a strong electron-withdrawing group (4-CF_3_) also afforded the desired product with an excellent yield. Additionally, 1-(1-naphthyl) ethanol and 1-(2-naphthyl) ethanol were converted to their corresponding acids in this reaction with high yields of 93.08% and 78.65%, respectively (Table 2, entries 9 and 10).

For the brominated substrates, some debrominated products were produced (Table 2, entries 13–15). For the 3-NH_2_-substituted substrate, the product was yielded at 76.26%. The thiobenzyl alcohol was oxidized to benzenesulfonic acid with a low yield of 20.97%. From these results, all benzyl alcohols with an electron-withdrawing and electron-donating group can be successfully transformed to the corresponding benzoic acids in moderate to excellent yields, but thiobenzyl alcohol cannot be successfully transformed by this catalytic system.

We also used this catalyst system with some other types of alcohols. 2-Phenylethanol, which is a primary alcohol with an alkyl group in one side, was oxidized to benzoic acid with 69.51% yield. For the 1-phenylethanol analogues, all were transformed to the corresponding acids in high to excellent yield. Oxidation of 1-(4-chlorophenyl)ethanol to the corresponding acid proceeded with 79.28% yield, but no dechlorination product was obtained.

It was clearly shown that the NaO^*t*^Bu/DMF catalytic system is inexpensive and efficient for the oxidation of 5-HMF and various alcohols to yield the corresponding acids. Next, to determine the reaction mechanism, several control experiments were carried out in which 2,6-di-*tert*-butyl-4-methyl phenol (BHT), a generally accepted radical scavenger in organic chemistry, was added to the reaction.^25^ It was shown that the addition of BHT clearly influenced the reaction. For example, a relatively low yield of FDCA was obtained after BHT (2.5 equiv.) was added (Table 3).

**Table 3:** Quenching experiments for the oxidation of benzyl alcohol

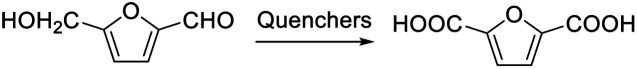			
Entry	Quenchers	Equivalents	Yield (%)
1	None	0	80.85
2	BHT	0.5	64.52
3	BHT	2.5	17.96

When we decreased the reaction time to 5 min, only 56.06% of 5-hydroxymethyl-2-furancarboxylic acid (HMCFA) and 12.36% of FDCA were obtained under standard reaction conditions (Table S2†). In this reaction, 5-HMF was not transformed to DCA but HMCFA; that is, the formyl group was easier to oxygenate to acid than the alcohol group under these catalytic conditions. We also added AIBN as a free radical initiator to this reaction,^26^ and the yield was increased to 89.33%, which indicated that the addition of AIBN efficiently promoted the reaction. These results indicated that the reaction might occur through the radical mechanism.

Thus, we propose a reaction mechanism as follows from the results and literature.^20^ When 5-HMF is used as the substrate, it will first be oxidized into HMCFA. Initially, the ^*t*^BuO^−^ (from NaO^*t*^Bu) was firstly rendered into single electrons with the assistance of O_2_ to offer ^*t*^BuO^.^ radicals. Then, the ^*t*^BuO^.^ radicals attacked the H atom in 5-HMF to produce intermediate I and ^*t*^BuOH. Intermediate I then rendered single electrons to O_2_ to offer immediate III.

Intermediate III then captured the H from ^*t*^BuOH to produce intermediate IV and regenerate a ^*t*^BuO^.^ single electron. Intermediate IV was added to 5-HMF to form the Criegee intermediate V, following by rearrangement to offer 2 mol of HMFCA salt. The HMFCA salt was further oxidized to FFCA, which repeated the above process to obtain the FDCA salt products. The FDCA salts were further acidified by HCl to afford the desired FDCA (Scheme 1).

**Scheme 1:**
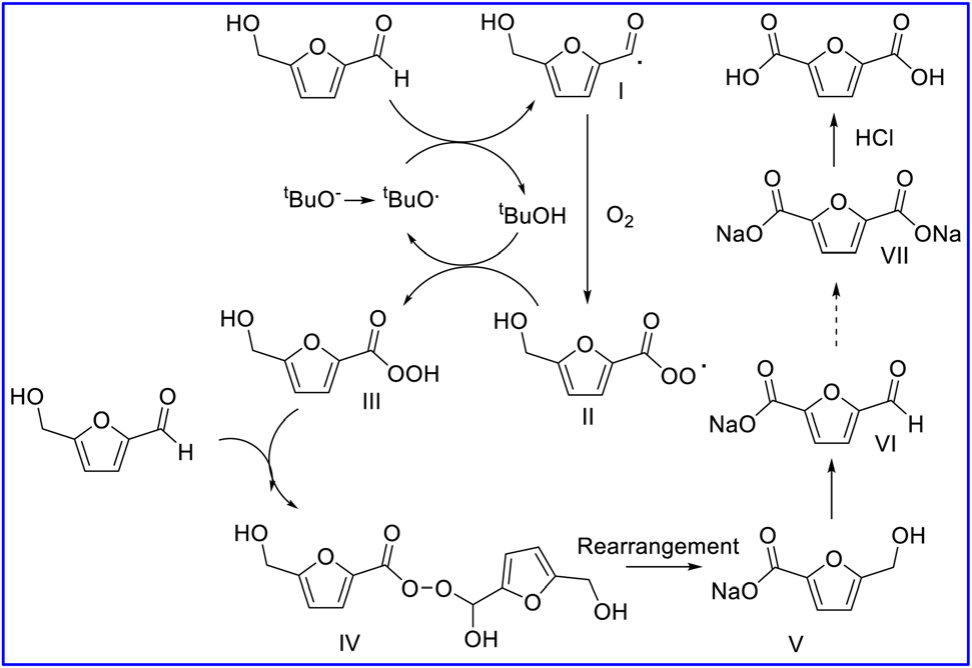
A proposed reaction mechanism.

In conclusion, we have developed an effective NaO^*t*^Bu/DMF catalytic system for the oxygenation of 5-HMF to FDCA. The catalytic system is simple and efficient, and is composed of a common base with an oxygen balloon in DMF. Moreover, some 5-HMF analogues and various types of alcohols were transformed to the corresponding acid in moderate to excellent yield. The greatest advantages of this catalytic system are its simplicity and ability to proceed under metal-free conditions. Thus, it is advantageous for biomass transformation and organic synthesis.

## Conflicts of interest

There are no conflicts to declare.

## Supplementary Material

RA-013-D3RA01104F-s001
