# Supplementary material for: Conversion of 5-hydroxymethylfurfural to furan-2,5-dicarboxylic acid by a simple and metal-free catalytic system
Source: RSC Adv. 2023 May 10;13(20):13819–23. doi: 10.1039/d3ra01104f (PMC10170353; doi:10.1039/d3ra01104f)

Electronic Supplementary Material (ESI) for RSC Advances.
This journal is © The Royal Society of Chemistry 2023

Conversion of 5-hydroxymethylfurfural to Furan-2,5-dicarboxylic Acid by a Simple and Metal-free Catalytic System

Xue Wang,[#,a] Xinyuan Guo ,[#,b] Xinmei Wang ,[#,b] Chi Li,[b] Shanjun Wang,[b] Han Li ,[b] Yan'an Gao,[c] Yiying Li, *[d] Jinhui Wang*[a,e] and Huanjun Xu*[b,e]

[a]Department of Medicinal Chemistry and Natural Medicine Chemistry, College of Pharmacy, Harbin Medical University, Harbin, China
[b]School of Science, Qiongtai Normal University, Haikou, 571127, China.
[c]Key Laboratory of Ministry of Education for Advanced Materials in Tropical Island Resources, Hainan University, Haikou 570228, China
[d]College of Basic Medicine and Life Sciences, Hainan Medical University, Haikou, China
[e]Key Laboratory of Child Cognition and Behavior Development of Hainan Province, Qiongtai Normal University, Haikou, China

*To whom correspondence should be addressed. E-mail: 15798946232@163.com

I. General experimental details

1.1. Materials

All of the materials were purchased from Beijing Innochem Company, and used as received.

1.2 Characterization

Some products were purified by flashchromatography on silica gel. Benzyl alcohols: analysis of crude reaction mixture was performed on a SHIMADZU 2030 GC System with a HPINNOWAX capillary column (30 m×0.25 mm×0.32 μm) and an FID detector. The following GC temperature program was used: 45°C is maintained for 2 minutes, rises to 280 °C at 15 °C/min, and hold for 5 minutes. Nitrogen was used as a carrier gas. The injector temperature was held at 250 °C.

5-HMF analogues: analysis of crude reaction mixture was performed on a SHIMADZU xx HPLC System with a C18 column (30 m×0.25 mm×0.32 μm). The following HPLC program was used: The 0.1% H_3PO_4 in CH_3OH/H_2O(70:30) as eluent and the detection wavelength was 284nm .

1.3 General procedures for aerobic oxidation

Typical procedure: Typical procedure: the desired amount of secondary alcohol substrate (0.5 mmol), NaO^{t}Bu(2 mmol), DMF (5 mL) were added into a 25 mL reaction bottle. Then the mixture

was degassed though three times with the oxygen balloon, the reaction was hold under 45 ^{o}C for the desired time. After being acidificated with 2 mol/L HCl (3 mL), the solution was extracted by ethyl acetate (5 mL) twice, the organic phase was washed with saturated brine once and dried by Na_2SO_4. The combined organic phase was removed the solvent by a rotary evaporator. The desired product was obtained through column chromatography using ethyl CH_2Cl_2/MeOH ether as eluent.

For GC analysis: After being acidificated with 2 mol/L HCl (3 mL), the solution was extracted by ethyl acetate (3 x 3mL). The combined substrate was centrifugated and then subjected to GC/FID for qualitative with dodecane as internal standard and for quantitative identification standard substrate.

For HPLC analysis: After being acidificated with 1 mL HCl, the solution was added methanol to 10 mL or 25 mL volumetric flask, then transferred 0.1mL to anther 10 mL volumetric flask, following by being subjected to HPLC for qualitative and quantitative by one point external standard method with standard substrate.

1.5 Table S1. The results of the oxidation of HMF into FDCA reported in literature

Table S1 The results of the oxidation of HMF into FDCA reported in literature

Entry	Metal	Base additive	Co-catalyst	Temp. (°C)	Time (h)	Oxide	HMF Conv./%	FDCA yield/%	Ref.
1	Au	Na_2CO_3 (4 equiv)	-	130	5	10 atm Air	100	96	7a
2	Pt	-	-	150	14	0.4 MPa O_2	99.9	98.6	8a
3	Pd	NaOH (1.25 equiv)	-	90	6	1 atm O_2	>99	78	9a
4	Ru-Co	-	-	150	24	10 bar O_2	100	99	10c
5	Ag-Au	-	-	90	2	5 atm O_2	100.0	95.5	11a
6	Mn	$NaHCO_3$ (2 equiv)	-	100	24	1 MPa O_2	>99	74	12a
7	Co	Cs_2CO_3 (1.5 equiv)		75	7	1 bar O_2	94.9	89.3	13a
8	Fe	-	TEMPO (1.0 equiv)	70	24	^{t}BuOOH (8 equiv)	>99	57	14a
9	Mo	NaOH (3 equiv)		100	24	1 MPa O_2	99	91	15a
10	-	K_2CO_3 (3 equiv)	NNC-99	80	48	1 bar O_2	99	80	16
11	-	NaOH (0.2 equiv)	I_2(0.1equiv)	70	10	TBHP (4 equiv)	-	53	18
12	-	NaO^{t}Bu	-	45	6 h	1 atm O_2	99.9	80.8	This work

1.4 Table S2. The products distribution with reaction time[a].

Reaction time	Yield of FFCA (%)	Yield of FDCA (%)	Conversion of HMF (%)
5 min	56.06	12.36	90.75%[b]
30 min	49.15	36.56	99.63%
1 h	37.67	47.74	99.72%
2 h	8.67	72.38	99.89%
3 h	3.95	76.20	99.85%
6 h	2.74	77.90	99.91%
9 h	2.67	77.32	99.76%

Condition: [a] substrate (0.5 mmol), NaO^{t}Bu (2.0 mmol), DMF (5 mL), O_2 balloon. [b] substrate (0.5 mmol), NaO^{t}Bu (2.5 mmol), DMF (5 mL), O_2 balloon

(1) Reaction solution (5 min) compared with 5-HMF standard[b]

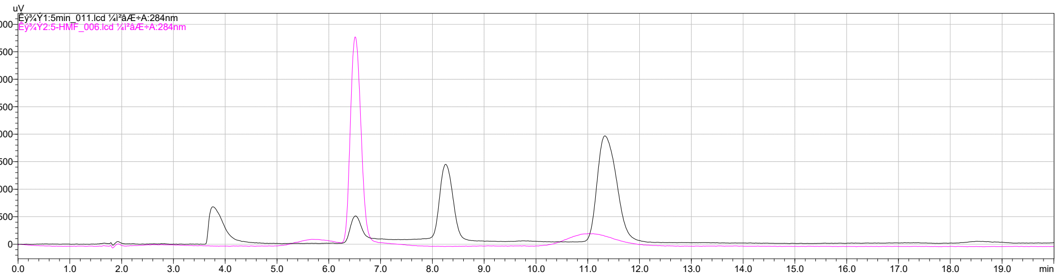

(2) Reaction solution (5 min) compared with DFCA standard[b]

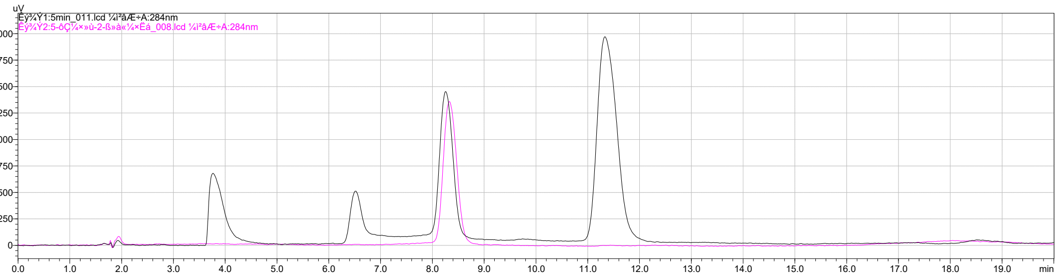

(3) Reaction solution (5 min) compared with DFF standard[b]

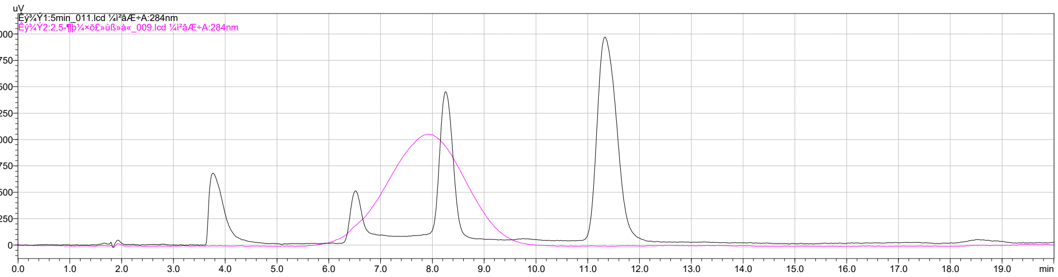

(4) Reaction solution (5 min) compared with FFCA standard[b]

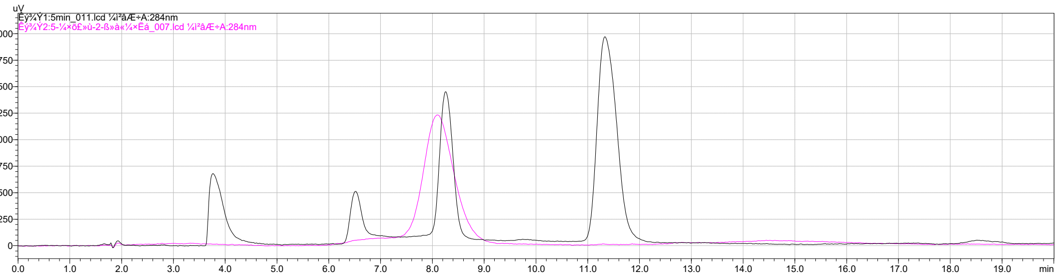

(5) Reaction solution (5 min) compared with FDCA standard[b]

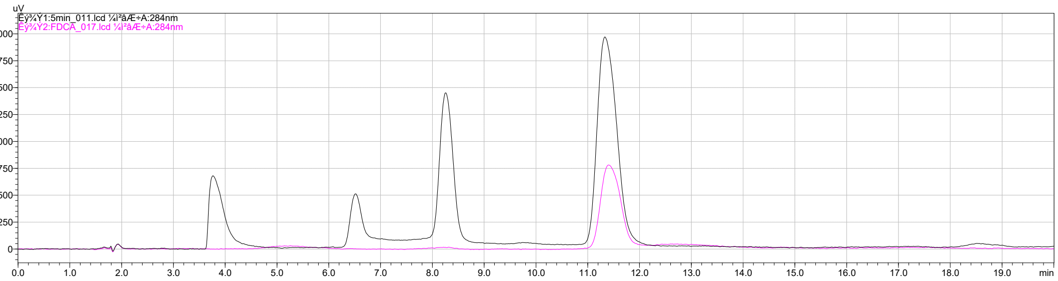

Supplement: RA-013-D3RA01104F-s001 [file RA-013-D3RA01104F-s001.pdf]
